# Supplementary material for: Effect of Vitamin D on Bone Regeneration: A Review
Source: Medicina (Kaunas). 2022 Sep 23;58(10):1337. doi: 10.3390/medicina58101337 (PMC9610653; doi:10.3390/medicina58101337)
Supplement: Supplementary file 1 [file medicina-58-01337-s001.zip › medicina-1898438-supplementary.pdf]

The search strategy was the following:
Search terms: (bone regeneration) AND (Vit. D)
("bone regeneration" [MeSH Terms] OR ("bone" [All Fields] AND "regeneration" [All Fields]) OR "bone regeneration" [All Fields]) AND ("Vit. D" [MeSH Terms] OR "Vit. D"[All Fields] OR" ergocalciferols "[MeSH Terms] OR" ergocalciferols "[All Fields])
Search terms: ("bone regeneration" [MeSH Terms] OR ("bone" [All Fields] AND "regeneration" [All Fields]) OR "bone regeneration" [All Fields]) AND ("Vit. D" [MeSH Terms] OR "Vit. D" [All Fields] OR "ergocalciferols" [MeSH Terms] OR "ergocalciferols" [All Fields]).
Search terms: (Vit. D) AND (osseointegration) AND (bone regeneration)
("Vit. D"[MeSH Terms] OR "Vit. D"[All Fields] OR "ergocalciferols"[MeSH Terms] OR "ergocalciferols"[All Fields]) AND ("osseointegrate"[All Fields] OR "osseointegrated"[All Fields] OR "osseointegrates"[All Fields] OR "osseointegrating"[All Fields] OR "osseointegration"[MeSH Terms] OR "osseointegration"[All Fields] OR "osseointegrative"[All Fields]) AND ("bone regeneration"[MeSH Terms] OR ("bone"[All Fields] AND "regeneration"[All Fields]) OR "bone regeneration"[All Fields])
